# Exploring autoantibody signatures in brain tissue from patients with severe mental illness

**DOI:** 10.1038/s41398-020-01079-8

**Published:** 2020-11-18

**Authors:** David Just, Anna Månberg, Nicholas Mitsios, Craig A. Stockmeier, Grazyna Rajkowska, Mathias Uhlén, Jan Mulder, Lars Feuk, Janet L. Cunningham, Peter Nilsson, Eva Lindholm Carlström

**Affiliations:** 1 Department of Protein Science, KTH Royal Institute of Technology, SciLifeLab, Stockholm, Sweden; 2 Department of Neuroscience, Psychiatry - Uppsala University, Uppsala, Sweden; 3 Department of Neuroscience, Karolinska Institute, Stockholm, Sweden; 4 Department of Psychiatry and Human Behavior, University of Mississippi Medical Center, Jackson, MS USA; 5 Science for Life Laboratory, Department of Immunology, Genetics and Pathology, Uppsala University, Uppsala, Sweden

**Keywords:** Molecular neuroscience, Pathogenesis, Scientific community

## Abstract

In recent years, studies have shown higher prevalence of autoantibodies in patients with schizophrenia compared to healthy individuals. This study applies an untargeted and a targeted affinity proteomics approach to explore and characterize the autoantibody repertoire in brain tissues from 73 subjects diagnosed with schizophrenia and 52 control subjects with no psychiatric or neurological disorders. Selected brain tissue lysates were first explored for IgG reactivity on planar microarrays composed of 11,520 protein fragments representing 10,820 unique proteins. Based on these results of ours and other previous studies of autoantibodies related to psychosis, we selected 226 fragments with an average length of 80 amino acids, representing 127 unique proteins. Tissue-based analysis of IgG reactivities using antigen suspension bead arrays was performed in a multiplex and parallel fashion for all 125 subjects. Among the detected autoantigens, higher IgG reactivity in subjects with schizophrenia, as compared to psychiatrically healthy subjects, was found against the glutamate ionotropic receptor NMDA type subunit 2D (anti-GluN2D). In a separate cohort with serum samples from 395 young adults with a wider spectrum of psychiatric disorders, higher levels of serum autoantibodies targeting GluN2D were found when compared to 102 control individuals. By further validating GluN2D and additional potential autoantigens, we will seek insights into how these are associated with severe mental illnesses.

## Introduction

In recent years, several studies have reported high autoantibody titers in patients with schizophrenia^1–3^. Additional studies have indicated increased levels of cytokines such as interleukin-1 (IL-1) and IL-6, as well as transforming growth factor-β, in schizophrenia patients during acute psychotic episodes with decreasing levels during treatment, while levels of interferon-γ, tumor necrosis factor-α, IL-12, and soluble IL-2 receptor remained elevated irrespective of clinical treatment^4,5^.

The concept that some cases of severe mental disorders such as schizophrenia might be caused by a chronic low-grade neuroinflammation was postulated by Bechter in the mild encephalitis hypothesis^6^. The identification of brain reactive autoantibodies with potential pathological relevance in cases presenting with primarily psychiatric symptoms such as psychosis has opened a whole new research field^7–9^. Diseases caused by autoantibodies binding to proteins in the central nervous system (CNS) are known as neuronal surface antibody (NSAb)-mediated diseases. It is of importance to be able to identify whether psychiatric patients suffer from NSAb-associated diseases since it will have a fundamental impact on the future for precision diagnosis and treatment as these patients usually respond well to immunotherapies^10^. NSAbs are implicated in diseases such as limbic encephalitis including *N*-methyl-d-aspartate (NMDA) receptor encephalitis^11,12^ where they bind to extracellular epitopes of NMDA receptors (NMDARs) causing a dysfunction, leading to psychosis and other neurological phenotypes in these patients^13^. Where early work focused on patients with severe neurological conditions such as epilepsy^14^, it is now clear that early stages of the disease may present a heterogeneous mix of psychiatric symptoms, such as anxiety, personality changes, and psychotic symptoms, including hallucinations and delusions^15^. The diagnosis of NMDAR encephalitis is currently based on the presence of GluN1 antibodies in the cerebrospinal fluid (CSF) of patients. However, a recent study has shown that GluN1 antibodies, independent of medical condition and immunoglobulin (Ig) class, are functional resulting in decreased GluN1 surface expression^16^.

Additional studies have shown that antibodies toward the NMDAR are also linked to psychiatric symptoms in neuropsychiatric systemic lupus erythematosus, where cross-reactivity between anti-DNA antibodies and the GluN2A subunit was reported^17^. Antibodies to extracellular epitopes of NMDARs and the voltage gated potassium channel complex (VGKC), specifically to epitopes of CASPR2 and LGI1, have been the subject of numerous studies^15,18,19^. Antibodies against VGKC may confer a clinical picture with psychotic symptoms, movement disorder, amnesia, and seizures^20,21^. The diagnosis of schizophrenia is currently based on a broad set of symptoms after exclusion of other known treatable diseases with similar clinical presentation. In patients with a clinical diagnosis of schizophrenia, the prevalence of known NSAbs is still rather low (approximately 4%), but this group of patients is extremely important to identify as other therapy options may be effective for these cases^22^. Whether or not these antibody interactions are relevant for the psychotic symptoms of these patients needs to be determined. Uncertainties regarding recognition and diagnosis of NSAb-mediated diseases were recently discussed by Pollak et al., and future diagnostic approaches for autoimmune psychosis were proposed^23^. These proposed criteria included that the patient has established psychotic symptoms of abrupt onset, movement disorders, adverse response to antipsychotics, and more. Proposed methods for investigation of autoimmune psychosis include electroencephalography, magnetic resonance imaging, serum autoantibodies, and CSF analysis.

NMDAR encephalitis is treated with immunotherapy and early intervention is crucial for improved clinical outcome^24,25^. This is an important argument for why early screening for pathogenic antibodies should be carried out at first presentation of associated symptoms^15^. Recently, the impact of potential pathogenic autoantibodies in postmortem brain tissue of affected patients has emerged. Hughes et al. examined postmortem hippocampal tissue of anti-NMDA-positive subjects and controls and found reduced NMDAR cluster density upon immunostaining with antibodies to the NR1 (GluN1) subunit of the receptor^26^. Additionally, a more recent study focused on the presence of endogenous IgG in postmortem brain tissue of subjects with schizophrenia and controls. The authors conclude that IgG antibodies are present in both schizophrenia subjects and controls and that brain-reactive antibodies are found in the blood of both groups^27^.

Anti-GluN1 in serum or CSF may be analyzed using both in vivo and in vitro methods, such as cell-based assays coupled to flow cytometry and enzyme-linked immunosorbent assays. Although commonly used cell-based assays enable expression and presentation of membrane proteins, they have limitations in terms of numbers of antigens that can be analyzed simultaneously and are thereby not optimal for explorative studies aiming to discover novel autoimmune targets. For this purpose, we have developed a microarray workflow, where autoantibody reactivity to 11,520 human protein fragments can be investigated simultaneously^28^. One of the aims with the broad screening approach was to increase the possibility to identify still unknown autoantibodies that may contribute to schizophrenia. As also suggested previously, autoantibody detection should be done in “panels” rather than investigating single autoantibodies because of the overlap between symptoms of autoimmune encephalitis and psychotic disorders, since several NSAbs may occur in individual patients^29^. Additionally, the already identified autoantibodies known to cause autoimmune encephalitis and psychiatric symptoms may only represent a tiny part of the whole picture. The antigens were generated within the Human Protein Atlas project (www.proteinatlas.org) as human protein fragments expressed in *Escherichia coli* with an average length of 80 amino acids containing sequences with low homology to other human proteins. Furthermore, we have used the suspension bead array technology^30^ to additionally explore IgG repertoires. We successfully used this technique in a previous study to widely explore the autoantibody repertoire in the healthy population^31^ and now aim to explore these repertoires in the context of severe mental illness. To our knowledge, this is the first study to use a systematic multiplexed affinity proteomics approach to profile the autoantibody repertoire in the brain tissue of subjects with schizophrenia.

## Methods

### Study population

Snap-frozen postmortem brain tissues from 73 subjects with schizophrenia and 52 controls were obtained from four different brain banks. We included 20 subjects with schizophrenia diagnosis and 20 neurologically healthy controls from the Harvard Brain Tissue Resource Center, 6 subjects and 12 neurologically healthy controls from MRC UK Brain Bank Networks, 27 subjects from the University of Mississippi Medical Center Brain Collection, and 20 subjects and 20 neurologically healthy controls from the London Neurodegenerative Disease Brain Bank (summarized in Table 1). Brain samples were obtained from the frontal cortex (Brodmann areas 8, 8 + 9, and 11 + 12). Schizophrenia subjects and controls were matched according to age, sex, and brain region (for a detailed information, see Supplementary Table 1 adapted from Moghadam et al.^32^). This table includes all available information on affection status, age, sex, postmortem interval (PMI), respective brain region, age of onset, use of antipsychotic drugs, brain pH, source of brain material, and cause of death. Informed written consent was obtained from legally defined next of kin for the tissue collection and is published in more detail by Moghadam et al. Ethical approval for the brain tissue samples was obtained from the Regional Ethic Review Board in Uppsala, Sweden (Dnr. 2012/082 and 2012/082/1).

**Table 1:** The table shows the sample overview of the brain tissue retrieved from the biobanks.

Brain bank	Schizophrenia [*n*] (M/F)	Classification system	Controls [*n*] (M/F)	Ave. age [years] (range)	Ave. PMI [h] (range)
Harvard Brain Tissue Resource Center	20 (10, 10)	Feighner	20 (10, 10)	62 (31–85)	23 (8–38)
MRC UK Brain Bank Networks	6 (5, 1)	DSMIV	12 (10, 2)	46 (36–70)	58 (35–96)
Mississippi Brain Collection	27 (15, 12)	DSMIV	NA	41 (23–65)	20 (6–74)
London Neurodegenerative Disease Brain Bank	20 (11, 9)	No information	20 (11, 9)	61 (31–90)	45 (7–100)

*Ave*. average, *F* females, *M* males, *n* number, *NA* not available.

Furthermore, the circulating autoantibody repertoire was studied in serum from 395 patients and 102 controls with multiple sampling for some individuals (143 total samples) from the Uppsala Psychiatric Patient (UPP) sample cohort^33^. Characteristics of patients and controls who donated the serum samples are included in Supplementary Table 2. Controls screening positive for mild or subclinical psychiatric conditions that did not require specialist psychiatric care now or previously were not excluded. Seven controls were taking low-dose antidepressants on a regular basis. One control was prescribed lamotrigine for epilepsy in childhood. Patients included in this study were new patients with severe psychiatric conditions aged 18–25 years from the “Young adults” section. Patients were diagnosed with primarily affective and anxiety disorders and five patients were found to have psychotic disorders. Diagnosis was based on the structured clinical interview for Diagnostic and Statistical Manual of Mental Disorders, Fourth Edition axis I disorders—clinical version or the mini-international neuropsychiatric interview^33^. Ethical approval for the UPP samples was obtained from the Regional Ethic Review Board in Uppsala, Sweden (Dnr. 2012/081 and 2014/148). Fully informed and written consent was obtained from each participant.

### Protein extraction

Snap-frozen tissue samples were molded in Optimal Cutting Temperature and 30 sections (10-µm thick) were sliced using a freezing microtome. Protein extraction was performed using Tissue Extraction Reagent I (Cat. No. FNN0071, ThermoFisher, Waltham, MA, USA) according to the manufacturer’s instructions. Briefly, prior to use the tissue reagent was supplemented with cOmpleteTM, Mini Protease Inhibitor Cocktail (Cat. No. 4693124001, Sigma-Aldrich, Darmstadt, Germany), to prevent protein degradation. The tissue was then homogenized in 100 µl Tissue Extraction Reagent with a pipette avoiding air bubbles, the homogenate was centrifuged at 10,000 × *g* for 5 min, and the supernatant was transferred to a new Eppendorf tube. The protein extract (denoted supernatant throughout the paper) was immediately frozen and stored at −80 °C until further use. The obtained tissue pellets were further processed to increase the possibility of eluting antibodies with high affinity to brain proteins. Antibody retrieval was performed using buffers of pH 3.0 (2.5% acidic acid) and 10.0 (NaOH). First, the pellets were re-suspended in 30 µl of low pH buffer and incubated for 20 min at room temperature (RT) using a tube shaker (internal mixer RM-2L, ELMI, Calabasas, CA, USA). The samples were then sonicated and incubated for an additional 20 min before the pellet and supernatant were separated by centrifugation at 14,000 rpm for 5 min. The supernatant was then transferred to a fresh tube, and the process was repeated with high pH buffer. Both supernatants were combined to obtain a pH neutral solution and the final preparation here is, throughout the paper, denoted as pellet.

### Planar microarray

To explore the repertoire of autoantibodies in human brain tissue, we used our in-house produced planar protein microarrays. The protein array consisted of 11,520 spotted protein fragments on a glass slide, representing 10,820 unique proteins. The protein fragments were printed onto functionalized glass slides using an array jet printer (Arrayjet, Edinburgh, Scotland) as described previously^28^. To assess tissue suitability for this method and for initial target selection, 5 randomly selected lysate patient samples were diluted 1:10 in a protein containing buffer (3% bovine serum albumin (BSA), 5% milk powder, 160 µg/ml His6ABP in 0.1% phosphate-buffered saline–Tween 20 (PBST)) and applied onto the microarray. After 2 h incubation, the slides were washed in 0.1% PBST and subsequently incubated with anti-His6ABP IgY-antibody (Immunotech HPA, Stockholm, Sweden, 1:40,000 in 0.1% PBST) at RT for 1 h. After additional washing, a secondary antibody mixture of goat anti-human IgG (a-hIgG, Alexa 647, Life Technologies, Carlsbad, CA, USA, 1:15,000 in PBST) and goat anti-chicken antibody (Alexa 555, Life Technologies, 1:15,000 in PBST) was used, and the microarray was incubated for an additional hour. After washing, scanning was performed using the Agilent Technologies Microarray scanner, and the slides were analyzed using the GenePix software.

### Suspension bead array

Autoantibody profiling was carried out using the previously described suspension bead array technology on tissue samples of 125 individuals^30^. Based on the planar array results, previous in-house screenings, and targets from literature, a total number of 226 protein fragments representing 127 proteins were selected for this study. The protein fragments with the length of about 80 amino acids were coupled to color-coded magnetic beads (MagPlex, Luminex Corp., Austin, TX, USA) as described elsewhere^34^. The coupled protein fragments were then mixed together to create a bead array in suspension. All extracted samples and lysates were first incubated with protein containing sample buffer (3% BSA, 5% milk powder, 160 µg/ml His6ABP in 0.1% PBST) in a dilution of 1:10 (1 h, shaking, RT). Diluted samples were then incubated with the suspension bead array for 16 h on a horizontal shaker at ambient temperature. The serum samples were diluted 1:250 in a protein containing buffer (3% BSA, 5% milk powder, 160 µg/ml His6ABP in 0.1% PBST) and incubated for 2 h at ambient temperature. Following incubation, all unbound antibodies were washed away using 3× 100 µl PBST (0.05% Tween) with an automated plate washer (EL406 Washer Dispenser—BioTek, Winooski, VT, USA). Subsequently, the beads were incubated with 50 µl secondary R-phycoerythrin-conjugated a-hIgG (Invitrogen, Carlsbad, CA, USA). After washing, bound autoantibodies were reported as median fluorescent intensities (MFIs) using a FlexMap 3D instrument (Luminex Corp.).

### Data analysis

For the planar microarray analysis, the scanned slides were further processed using the GenePix software (Molecular Devices, CA, USA), and the fluorescence intensity of each spot was reported as a median value. Reactivity for each sample was defined as 20 times the median absolute deviation (MAD) plus the median of each individual sample over the whole data set as described previously^31^. For the suspension bead array, the median of each individual sample across the MFI of each antigen was calculated together with the MAD for each data point in the profile. The cutoff for reactivity was set to 15× MAD plus the median intensity in each individual sample. For the correlation analysis, all raw data points obtained were used and a Spearman’s rho correlation coefficient was calculated. All statistical calculations and visualizations were done using R and R studio^35^. For group comparisons, the data were converted into a binary variable based on the defined cutoff and *p* values were calculated using Fisher’s exact test. The web browser-based tool Protter was used for visualization of protein structures and features^36^.

## Results

### Exploring the autoantibody repertoire in human brain tissue

In this study, the autoantibody repertoire of subjects with schizophrenia was profiled using postmortem brain tissues. For a discovery approach, we utilized our in-house-produced protein fragment microarrays including 11,520 antigens and explored the presence of autoantibodies in brain lysates from 5 randomly selected subjects with schizophrenia. The individuals displayed reactivities to only a few individual antigens in this set-up, ranging from zero fragments for one patient, one fragment for two patients, three fragments for one patient, and ten protein fragments for one patient. In total, we found 14 reactive antigens that all were observed in single subjects only (Supplementary Table 3).

**Fig. 1:**
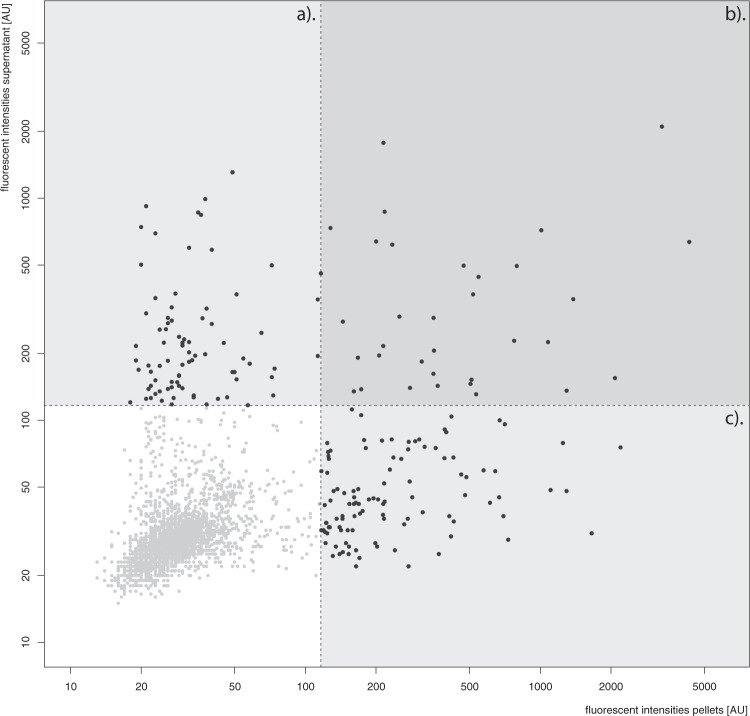
Correlation of all obtained fluorescent intensities from the suspension bead array using one sample from the brain supernatant (lysate) preparation and one from the brain tissue pellet preparation. Shown are only antigens that displayed reactivity in at least one sample type. **a** Reactive in only supernatant (*n* = 80), **b** reactive in both (*n* = 33), **c** reactive only in pellets (*n* = 110).

As the next stage, we profiled the autoantibody reactivity in all 125 brain samples on 226 different protein fragments (representing 127 proteins) using the suspension bead arrays. An overall comparison of reactivity between the sample preparation methods, denoted supernatant and pellet, respectively (see “Methods”) showed that, although the intensity levels were similar between the brain lysate supernatant and extracted lysate pellets (*r* = 0.48), the two sample types generated largely complementary reactivity profiles (Fig. 1). However, comparing all data obtained from the supernatants and pellets on an individual basis indicated that concordant reactivities could also be identified (Supplementary Fig. 1).

### Autoreactive antibodies in human brain tissues

**Fig. 2:**
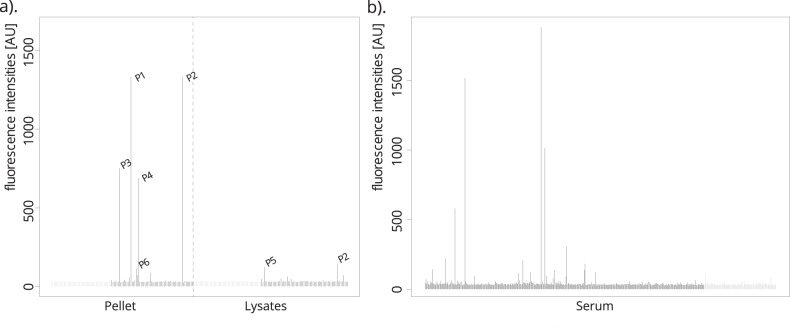
Detected levels of IgG reactivity toward the GluN2D protein. IgG reactivity toward the GluN2D protein fragment in brain tissue lysates from the pellet and supernatant preparations of the six schizophrenia patients that showed higher GluN2D reactivity compared to the other positive autoantibodies (**a**) and serum from patients with a range of mental illnesses (**b**). Controls samples are colored in gray, patient samples in black.

A first exploratory analysis revealed that a total number of 73 out of 226 protein fragments (32%) showed at least one reactive sample. Further we observed that most of the samples were reactive toward one or two antigens (Supplementary Fig. 2). By further characterizing reactivities in both subjects and control samples, we found that autoantibodies binding to the glutamate ionotropic receptor NMDA type subunit 2D (GluN2D) were present more frequently in subjects compared to controls (one-sided *p* = 0.01, Fig. 2a). In total, 8 of the 73 subjects with schizophrenia had reactive antibodies in their brain tissue, while none of the 52 control samples showed reactivity toward GluN2D.

**Fig. 3:**
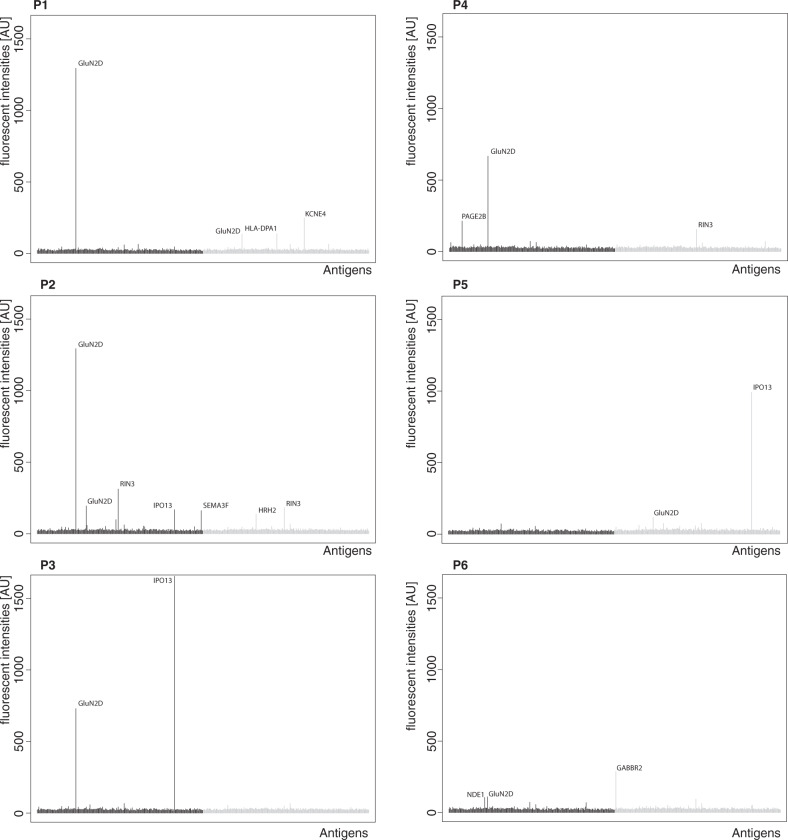
Individual profiles of six reactive schizophrenia subject samples (P1–P6), showing either the highest or second highest reactivity toward GluN2D compared to other positive antigens. Results from the pellet preparations are colored in black and results from the supernatant preparations are colored in gray.

**Fig. 4:**
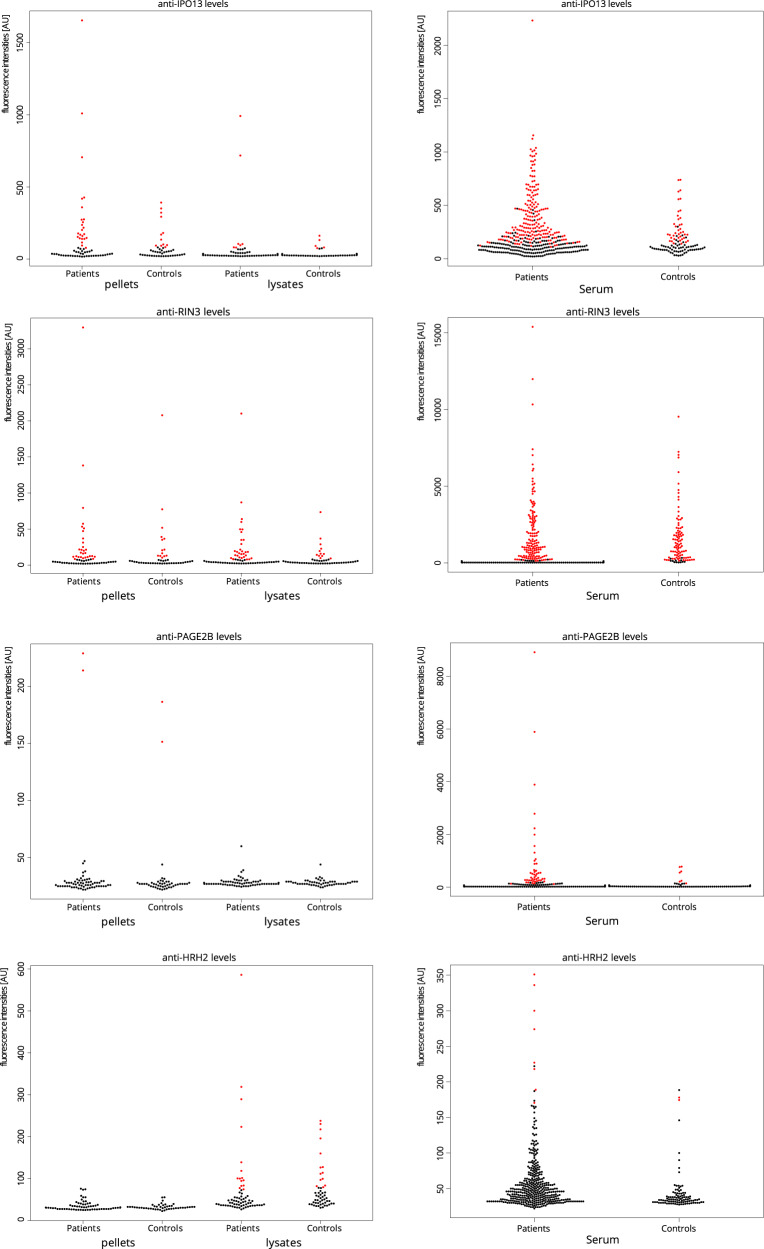
IgG reactivity toward four additional protein fragments identified in GluN2D-reactive samples with more than single reactivities. IgG reactivity from both pellet and supernatant preparations, as well as IgG reactivity of serum samples, are shown. Individual samples colored in red met the corresponding cut-off criteria for positive reactivity.

The eight reactive subjects with schizophrenia were further characterized. Six of the subjects revealed that GluN2D reactivity generated the highest or second highest intensity compared to other positive antigens. The data for those six samples is summarized in Fig. 3. Other identified multiple reactive antigens, among GluN2D-reactive samples were IPO13 (Importin-13), RIN3 (Ras and Rab interactor 3, *p* = 0.03), PAGE2B (Putative G antigen family E member 3), and HRH2 (histamine receptor H2). Both IPO13 and RIN3 showed a general high reactivity among all samples, while PAGE2B reactivity was observed in two schizophrenia subjects and two controls. HRH2 reactivities were observed in 15 schizophrenia subjects and 14 control samples (Fig. 4 and Tables 2 and 3). No reactivity was observed to the other NMDAR subunit protein fragments included in this study (GluN1, GluN3B). We further examined potential relationships between PMI, age, and brain pH and the respective GluN2D autoantibody reactivity, but there were no statistically significant associations.

**Table 2:** The table lists the reactive antigens identified in brain tissue from six schizophrenia subjects.

Gene name	Protein name	Uniprot	Antigen #	Fisher’s *p* value (one sided)	Reactive individuals (case/control)
*HRH2*	Histamine receptor H2	P25021	HPRR2090039	0.5	15/14
*IPO13*	Importin 1	O94829	HPRR2290067	0.2	31/17
*GRIN2D*	Glutamate ionotropic receptor NMDA type subunit 2D	O15399	HPRR2640053	0.01	8/0
*RIN3*	Ras and Rab interactor 3	Q8TB24	HPRR2970028	0.03	59/26
*PAGE2B*	PAGE family member 2B	Q5JRK9	HPRR3340274	1	2/2

**Table 3:** The table shows the gene name and the amino acid sequence of the protein fragments with reactivity in the brain tissue.

Gene name	Antigen seq (aa)
*HRH2*	NRDFRTGYQQLFCCRLANRNSHKTSLRSNASQLSRTQSREPRQQEEKPLKLQVWSGTEVTAPQGATDR
*IPO13*	KVLKCFSSWVQLEVPLQDCEALIQAAFAALQDSELFDSSVEAIVNAISQPDAQRYVNTLLKLIPLVLGLQEQLR QAVQNGDMETSHGICRIAVALGENHSRALLDQVEHWQSFLALVN
*GRIN2D*	LHRYFMNITWDNRDYSFNEDGFLVNPSLVVISLTRDRTWEVVGSWEQQTLRKYPLWSRYGRFLQPVD
*RIN3*	AKKNLPTAPPRRRVSERVSLEDQSPGMAAEGDQLSLPPQGTSDGPEDTPRESTEQGQDTEVKASDPHSMPEL PRTAKQPPVPPPRKKRISRQLASTLPA
*PAGE2B*	RSQSSERGNDQESSQPVGSVIVQEPTEEKRQEEEPPTDN

### Autoreactive antibodies to GluN2D in the serum

We further analyzed GluN2D antibody reactivity and associations in serum samples from a cohort of patients with a wide spectrum of psychiatric disorders. Following the observation of elevated anti-GluN2D presence in the brain tissue, we discovered that GluN2D reactivity was more frequent in 395 serum samples of young adults with a wide spectrum of psychiatric disorders compared to controls. In this analysis, we found eight patients and no control individuals with reactivity toward GluN2D (*p* = 0.03; Fig. 2b). For the eight patients, there is no clear clinical commonality. In a structured diagnostic interview (mini-international neuropsychiatric interview), six of the eight patients met the criteria for current depressive episode, one for bipolar disease type II, one for alcohol abuse, and the remaining two patients reported severe anxiety disorders. At sampling, five patients were unmedicated, two patients were prescribed antidepressants, and one patient was prescribed mesalamine, lamotrigine, and quetiapine. One of the eight patients had been diagnosed with irritable bowel disease while the remaining patients had no known somatic disorders. We further explored reactivities toward IPO13, RIN3, PAGE2B, and HRH2 in the serum samples (Fig. 4). Both PAGE2B and HRH2 showed a higher frequency of reactivity in patients.

## Discussion

This study applied affinity proteomic tools to identify and explore potential disease-associated autoantibodies in patients with schizophrenia. Autoantibody reactivity toward the NMDAR subunit 2D (GluN2D) was identified in 11% of postmortem tissue samples and in none of the control individuals suggesting a potential association with the disease. Beyond the diagnosis of schizophrenia, high level of autoantibody in brain tissue was not associated with tissue quality, cause of death, or the other available clinical factors.

A functional NMDAR consists of heterotetramers of subunits GluN1, GluN2, and GluN3 family proteins encoded by *GRIN1*, *GRIN2*, and *GRIN3* genes, respectively^37,38^. However, receptor trafficking from endoplasmic reticulum to the cell surface is dependent on subunit assembly and most receptors consist of a GluN1 subunit and one or multiple GluN2 subunits where GluN2A and GluN2B represent the majority of subunits found in functional receptors, whereas GluN2D is mainly expressed in early development^39^. Additionally, NMDAR surface mobility greatly depends on the GluN2A and GluN2B subunits^40^. A recent study evaluated the NMDA distribution and mobility along the synapse in the presence of anti-NMDA antibody-positive patient serum samples. Upon introducing NMDA autoantibodies, the distribution of GluN2A subunits along the synapse was reduced^41^. Since GluN2D is reportedly expressed mainly during development, we investigated the sequence homology between these subunits. As shown in Supplementary Fig. 3, Glun2D has a high sequence identity toward GluN2A (indicated by red diamonds with blue center) for the herein targeted protein fragment. A typical antibody recognition site is about five amino acid residues and does not need to be a continuous epitope. However, 60% of discontinuous epitopes have at least 3 amino acids in a continuous stretch^42^ at the same time as modified single amino acids can be sufficient as an antibody-binding epitope^43^. An approach to further investigate the potential binding site of the studied protein–autoantibody interaction is to use a suspension bead array with overlapping peptides covering the herein utilized protein fragments to map the corresponding binding epitope of the antibody^44^.

The vast majority of studies on autoantibodies in psychiatric disorders included serum samples. In one recent study, including 121 patients affected with schizophrenia and 230 control individuals, antibodies targeting NMDAR were measured in the serum and CSF. The results showed a statistically significant difference between the patient and control groups, where 9.9% of the patients showed increased prevalence of different types of NMDAR antibodies while only 0.4% of controls were seropositive^45^. The percentages are similar to the findings in our study and are higher compared to other studies in serum samples, usually the frequency of patients showing autoantibody reactivity is around ≤5%, as reviewed previously^46^.

For the majority of the samples in our study, the highest reactivity was measured in the processed tissue pellet which might indicate that reactive autoantibodies are bound to targets located in the brain tissue rather than bound to targets in residual blood components that might have diffused into the brain after death. By determining the prevalence and levels of specific autoantibodies with the approach used in our study, the role of autoantibodies in the development of psychiatric disorders such as schizophrenia may be elucidated.

To follow-up on our finding, we have screened serum of a cohort of young adults remitted to psychiatry specialists for more debilitating forms of mental illness. Notably, these patients are aged between 18 and 25 years, and in early stages of disease, more than half of the patients were untreated. Eight patients, of whom seven were women, were found to have serum GluN2D reactivity in the young adult population. As mentioned above, these patients had a wide range of diagnosis; however, restlessness, depression, and anxiety are very common first signs of psychotic disorders, and the prodromal phase may extend over years before the positive symptoms emerge^47^. A more thorough characterization and a follow-up study on these patients are required to draw any further conclusions. The frequency of reactive patients found in this study is relatively low but similar to those reported elsewhere for disease-associated antibodies^15,22^.

Each individual has a variety of natural occurring antibodies in their blood. A current report showed no evidence of a widespread antibody dysregulation of IgGs in the brain tissue among affected patients when they measured the general level of IgGs in the brain tissue using western blot and immunohistochemistry. The authors concluded that the methods used in their study may not have been appropriate to detect alterations of specific antibodies^27^. Autoreactive antibodies involved in the disease pathology may not affect the overall IgG levels but could still be important for the disease development or reflect disease process.

These antibodies could also have been triggered by exogenous antigens, such as bacterial or viral antigens that potentially could cross-react with brain antigens^48^. In the literature, naturally occurring antibodies have been widely studied in different aspects and are considered to be essential components of the immune response^49^. It has been observed that these antibodies are individual specific and similar levels are maintained over time, which lead to the hypothesis of an autoantibody fingerprint^31,50^. It is possible that differences in symptoms and severity in patients with GluN2D autoantibodies are related to the degree of damage to the blood–brain barrier and/or other factors such as differences in strength of antibody affinity to the receptor. It is also possible that these antibodies are co-occurring with another risk factor for psychiatric disease.

There are limitations regarding the analysis of brain-associated autoantibodies in the context of psychiatric conditions. In many cases, terminal illness often includes breaches of the integrity of the blood–brain barrier where pathological processes take place in the brain that may not have been ongoing prior to the terminal event^48^. In addition, the accessibility to specific disease-related tissue material of high quality and an appropriate translational animal model is limited. Additional in vitro models, such as cultured neurons to study impact of brain-reactive autoantibodies and corresponding protein expression and organization, provide us with valuable insights into the disease but may not represent the true disease state in its entirety. Cell-based assays are useful tools for the diagnosis of potential autoimmune disease and are successfully used for targets such as GluN1 (NR1), dopamine D2 receptor (DR2), and myelin-oligodendrocyte glycoprotein. Limitations to these techniques, however, are their low throughput, low grade of standardization, and that only extracellular conformational epitopes can be detected^51^. Nevertheless, these techniques, together with others, are valuable tools to detect and study brain reactive autoantibodies in less invasive sample materials, such as plasma/serum and CSF.

In this study, we have explored the IgG repertoires of postmortem brain tissues derived from a cohort of schizophrenia patients. We have explored these repertoires using affinity-based proteomic techniques revealing potentially interesting findings for the research community. However, our study has several limitations. First, our findings are based on a single detection method and the study lacks further analysis with orthogonal detection methods, including, e.g., cell-based assays, immunohistochemistry, or commercially available immunoassays.

Second, our study is primarily based on postmortem brain tissue, a sample material not suitable for routine clinical analysis. To address this, we also analyzed a second cohort of young patients displaying a wide range of psychiatric symptoms. However, none of the GluN2D-positive patients in the serum cohort had signs of schizophrenia. This could indicate that GluN2D reactivity is not specific for a schizophrenia diagnosis but rather may represent a yet unknown mechanism or more general vulnerability factor for psychiatric disorders. It is also possible that the presence of anti-GluN2D in serum may have different significance than antibodies in the CNS; CSF samples were unfortunately not available for analysis in the young adult cohort. Psychiatric symptoms are common when screening the general population, and we have chosen to include individuals within control population with current and previous mild psychiatric symptoms and medication with low-dose antidepressants. These control individuals, however, do not share the symptom severity or loss of function found in patients in this cohort who were referred for specialist psychiatric care.

Third, the obtained postmortem tissues were selected from four different brain banks and were provided with a variety of clinical information, and for many of the samples, information on duration of illness and medication as well as brain pH is missing. This also includes the lack of information regarding potential age-related disorders, including neurodegeneration. Additionally, for the majority of the obtained samples the PMI was relatively large, and subsequent protein degradation affecting antibody detectability cannot be excluded.

In summary, we have profiled the antibody repertoire in brain tissue and serum and identified elevated levels of antibodies against the GluN2D subunit of the NMDAR complex in a subgroup of subjects with schizophrenia and young adult patients with a wider spectrum of early stage psychiatric disorder using an affinity proteomics approach. Continued exploration and characterization of these patients as well as identified autoantibody targets may improve the understanding of psychiatric illnesses and in particular the involvement of autoantibodies to neuronal antigens.

## Supplementary information

Supplementary text

Supplementary Figure 1

Supplementary Figure 2

Supplementary Figure 3

Supplementary Table 1

Supplementary Table 2

Supplementary Table 3
