# Supplementary material for: Exploring autoantibody signatures in brain tissue from patients with severe mental illness
Source: Transl Psychiatry. 2020 Nov 18;10:401. doi: 10.1038/s41398-020-01079-8 (PMC7676257; doi:10.1038/s41398-020-01079-8)
Supplement: Supplementary file 1 — Supplementary text [file 41398_2020_1079_MOESM1_ESM.docx]

**Supplementary information for** ” Exploring autoantibody signatures in brain tissue from patients with severe mental illness”

Corresponding author:

Dr. Eva Lindholm Carlström

Department of Immunology, Genetics and Pathology, Medical Genetics and Genomics, Uppsala University

Sweden

Phone: +46 18 4714855

Email: [eva.lindholm@igp.uu.se](mailto:eva.lindholm@igp.uu.se)

This supplementary file includes:

1) additional information regarding the brain samples collected from Harvard Brain Tissue Resource Center, MRC UK Brain Bank Networks, University of Mississippi Medical Center Brain Collection and from the London Neurodegenerative Disease Brain Bank. For detailed information regarding each patient, see Supplementary table 1.

2) Supplementary legends for figures and tables included in the manuscript.

**Additional information regarding the brain tissue samples:**

Harvard Brain Tissue Resource Center:
Data on donors is extracted from health records and an extensive questionnaire completed by the family as well as from a neuropathology report. All tissue diagnoses are confirmed by retrospective review of clinical records and a comprehensive neuropathological examination.

MRC UK Brain Bank Networks:

The majority of the schizophrenia cases were collected in the 1990's through a collaboration between the biobank and a local psychiatrist that met the patients in the clinic. Basic clinical information was obtained by the biobank from the psychiatrist and from medical records, along with the neuropathological assessment.

University of Mississippi Medical Center Brain Collection:

The Institutional Review Boards of the University of Mississippi Medical Center, Jackson, MS, and the University Hospitals Cleveland Medical Center, Cleveland, OH, approved all procedures in accordance with the Declaration of Helsinki. Informed consent was obtained from legally-defined next-of-kin for the collection of tissue, medical records, and interviews. A master-level social worker administered the Structured Clinical Interview for DSM-IV Axis I Disorders (First et al., 2002) to knowledgeable informants as previously described (Zhu et al., 2012). Psychopathology of the subjects was determined by a board-certified clinical psychologist and a board-certified psychiatrist who independently reviewed the diagnostic interview scoring notations, the medical examiner's report, and any prior medical records. A consensus on the diagnosis was reached by the social worker, the clinical psychologist and the psychiatrist. Subjects selected for the study met diagnostic criteria for schizophrenia or schizoaffective disorder according to the Diagnostic and Statistical Manual of Mental Disorders IV (DSM-IV, 4th ed, 1994). Subjects were not included if there was any evidence of a neurological disorder, or gross or microscopic neuropathology. The medical examiner’s office determined the presence of psychotropic medications and substances of abuse in blood and urine. Tissues were obtained from the Human Brain Collection Core (Center for Psychiatric Neuroscience, University of Mississippi Medical Center, Jackson, Mississippi). Tissues were collected at autopsy at the Cuyahoga County Medical Examiner's Office, Cleveland, OH, and the Medical Examiner determined the cause of death. Samples of right prefrontal cortex (Brodmann area 8/9) were dissected without fixation, rapidly frozen in 2-methylbutane cooled in powdered dry ice, and transported in dry ice to storage at −80 °C.

London Neurodegenerative Disease Brain Bank:

All the data provided to us from the biobank is included in Supplementary Table 1.

References:

American Psychiatric Association (APA) (1994). Diagnostic and statistical manual of mental disorders. 4th ed. Washington: APA.

First M, Spitzer R, Gibbon M, Williams J (1995) Structured clinical interview for the DSM-IV Axis I disorders (SCID patient edition), version 2.0. New York, NY: New York State Psychiatric Institute.

Zhu H, Urban DJ, Blashka J, McPheeters MT, Kroeze WK, Mieczkowski P, Overholser JC, Jurjus GJ, Dieter L, Mahajan GJ, Rajkowska G, Wang Z, Sullivan PF, Stockmeier CA, Roth BL. Quantitative analysis of focused a-to-I RNA editing sites by ultra-high-throughput sequencing in psychiatric disorders. PLoS One. 2012;7(8):e43227. Epub 2012 Aug 17.

**Legends for supplementary figures and supplementary tables included in the manuscript:**

Supplementary Figure 1:

Correlation analysis of four reactive GluN2D patients (P1-P4). All reactive antigens are labeled with the respective antigen name.

Supplementary Figure 2:

Reactivity overview of a). number of antigens with at least one reactive sample and b). number of individuals and corresponding number of antigens. Samples derived from pellets are colored black, samples derived from supernatants are colored grey.

Supplementary Figure 3:

Predicted protein sequence of GluN2D showing the reactive protein fragment [360-427] in blue and the sequence identity towards GluN2A with red diamonds with blue center.

Supplementary Table 1:

The table summarizes the data obtained from the biobanks regarding the patients and samples, such as age, sex, PMI, brain region, age of onset, antipsychotic drugs, brain pH, brain bank and cause of death. For samples obtained from University of Mississippi Medical Center Brain Collection medications ever taken are listed in lower case and those prescribed in the last month of life are in all caps.

Supplementary Table 2:

Characteristics of the Uppsala Psychiatric Patients cohort. P-values are based on results from chi-square test (or Fisher’s when applicable).

Supplementary Table 3:

Reactive protein fragments detected in five different patient samples using a 11520 protein fragment array. Information includes gene name, protein name, uniprot ID, protein fragment identifier (antigen #) and protein fragment sequence (antigen seq (aa)).
