# Supplementary figures and images for: Exploring autoantibody signatures in brain tissue from patients with severe mental illness
Source: Transl Psychiatry. 2020 Nov 18;10:401. doi: 10.1038/s41398-020-01079-8 (PMC7676257; doi:10.1038/s41398-020-01079-8)

**P 1**

Spearman's Rho = 0.788

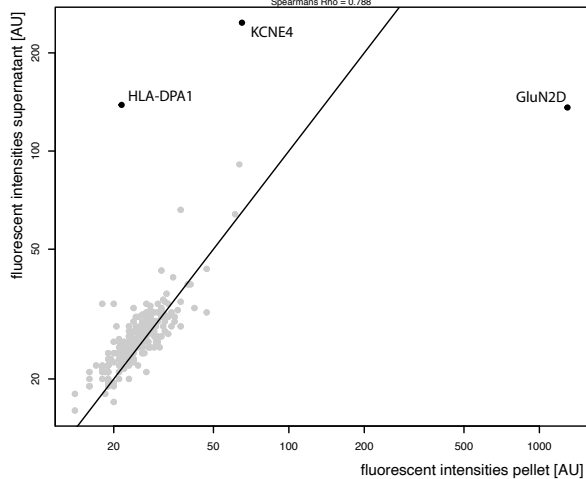**P 2**

Spearman's Rho = 0.755

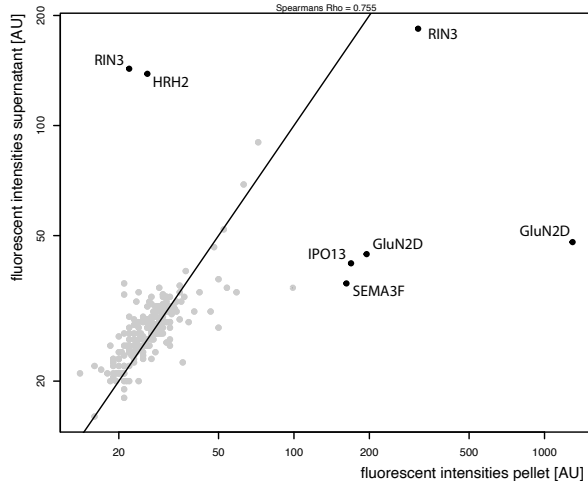**P 3**

Spearman's Rho = 0.704

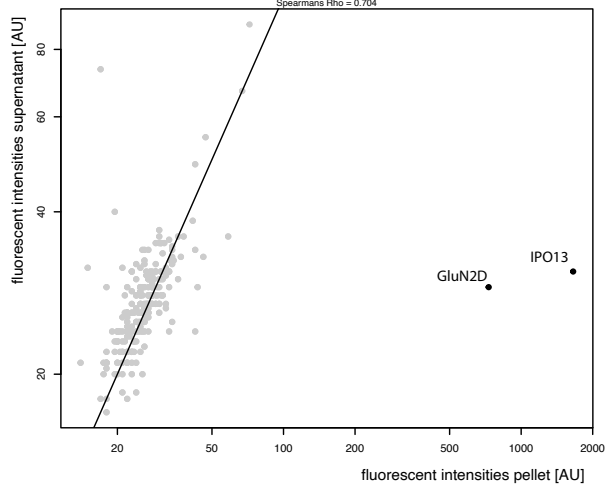**P 4**

Spearman's Rho = 0.764

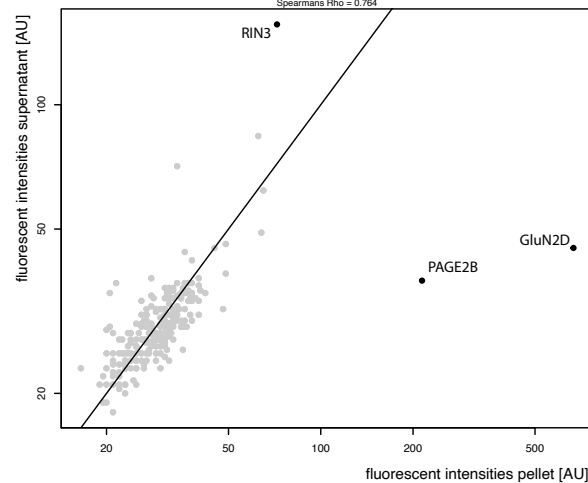

Supplement: Supplementary file 2 — Supplementary Figure 1 [file 41398_2020_1079_MOESM2_ESM.pdf]

a).

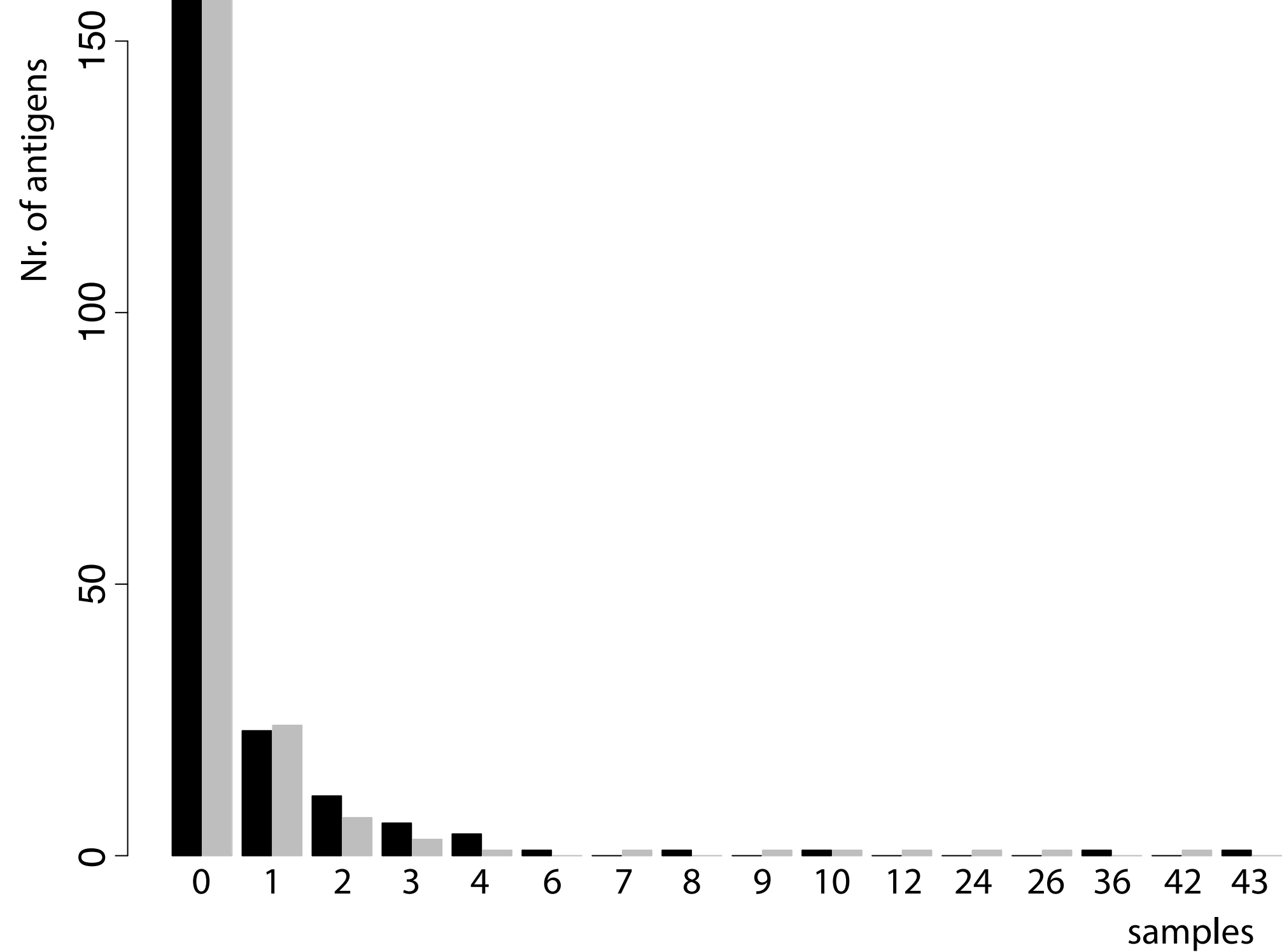

b).

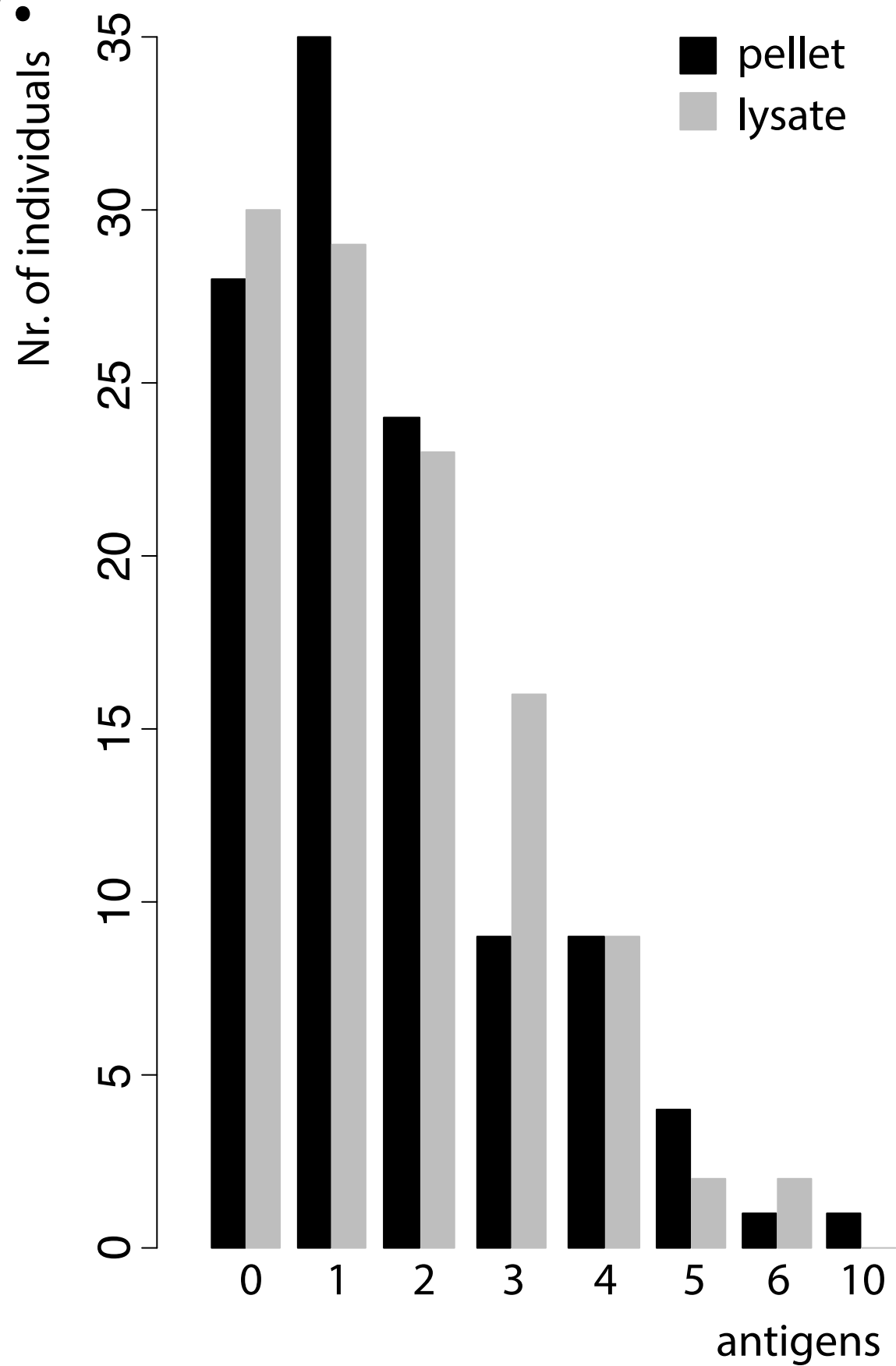

Supplement: Supplementary file 3 — Supplementary Figure 2 [file 41398_2020_1079_MOESM3_ESM.pdf]

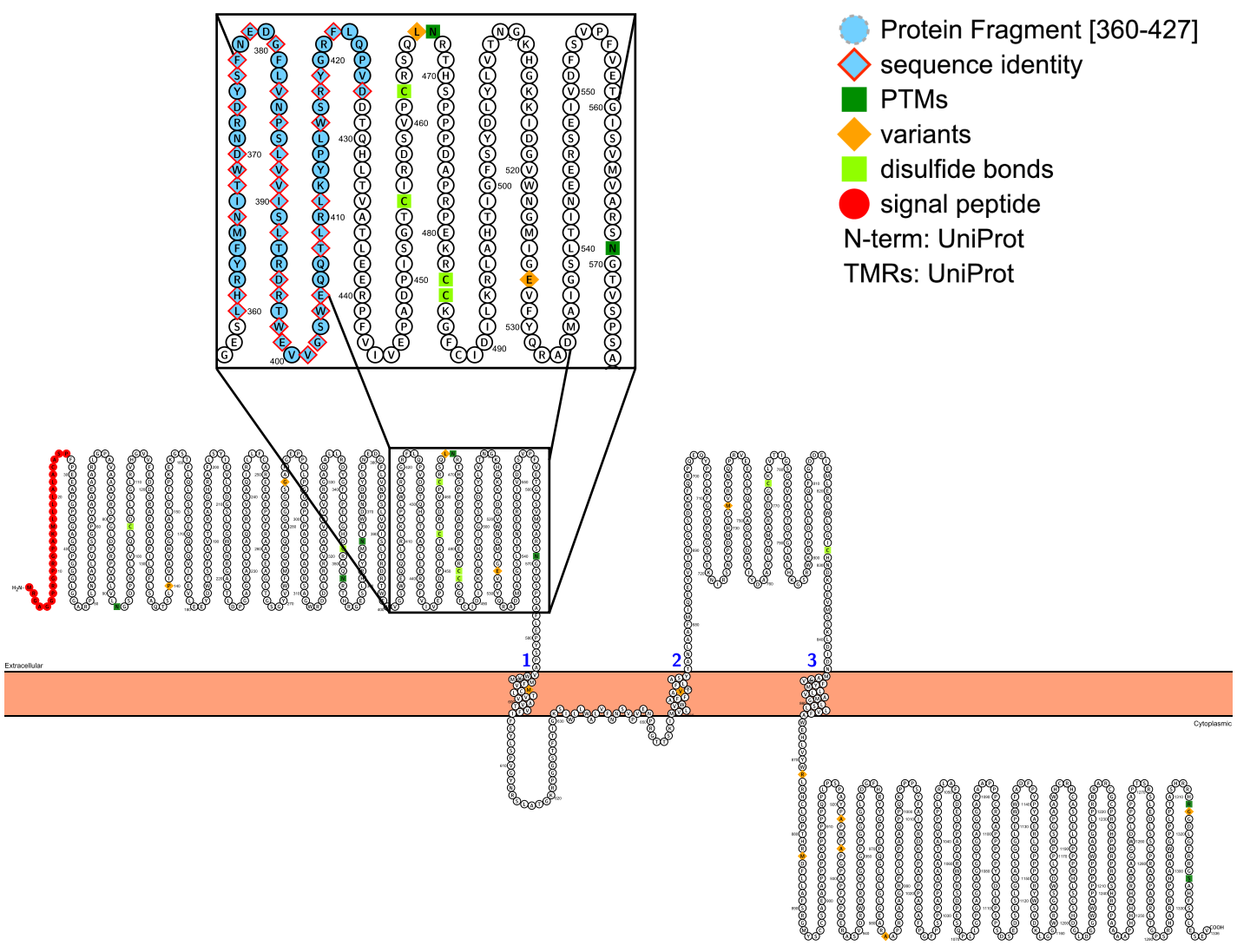

Supplement: Supplementary file 4 — Supplementary Figure 3 [file 41398_2020_1079_MOESM4_ESM.pdf]
