# Supplementary material for: Exploring autoantibody signatures in brain tissue from patients with severe mental illness
Source: Transl Psychiatry. 2020 Nov 18;10:401. doi: 10.1038/s41398-020-01079-8 (PMC7676257; doi:10.1038/s41398-020-01079-8)
Supplement: Supplementary file 5 — Supplementary Table 1 [file 41398_2020_1079_MOESM5_ESM.pdf]

Sample #	Brain bank ID	Affection status	Age	Sex	PMI (hrs)	Brain region	Age of onset	Antipsychotic drugs	Brain pH	Brain Bank	Cause of death
S1	AN07790	Scz	59	M	27	BA 8	No Info.	Clozapine 100 mg-once daily	No Info.	HBTRC	Natural and Cause of death-Colon Cancer
S2	AN08951	Scz	69	F	20	BA 8	No Info.	Zyprexa 10 mg-once daily	No Info.	HBTRC	Natural and Cause of death-Cardiac Arrest
S3	AN08082	Scz	58	F	21	BA 8	No Info.	Haloperidol 5 mg every 6 hrs as needed	No Info.	HBTRC	Natural and Cause of death-Myocardial Infarction
S4	AN12408	Scz	58	M	37	BA 8	No Info.	Risperdal 2 mg-once daily	No Info.	HBTRC	Natural and Cause of death-Atherosclerotic Heart disease
S5	AN12312	Scz	60	M	34	BA 8	No Info.	Zyprexa 20 mg and Abiflify 2 mg-once daily/Thorazine, Haldol, Lithium Carbonate-unknown dosages	No Info.	HBTRC	Natural and cause of death-Chronic Obstructive Pulmonary Disease
S6	AN12940	Scz	66	M	22	BA 8	No Info.	Zyprexa 20 mg -once daily, FazaClo 25 mg-three times daily, Prochlorperazine and Geodon-unknown dosage	No Info.	HBTRC	Natural and Cause of death-Advanced metastatic cancer of gastroesophageal junction
S7	AN03019	Scz	47	F	32	BA 8	No Info.	Invega 6 mg once daily and Risperidone 0.5 mg every 6 hours as needed	No Info.	HBTRC	Natural and Cause of death-Dilated Cardiomyopathy
S8	AN19868	Scz	79	F	8	BA 8	No Info.	No Info.	No Info.	HBTRC	Natural and Cause of death-Respiratory Failure
S9	AN03098	Scz	32	M	38	BA 8	No Info.	Risperdal 3 mg-twice daily, Abilify	No Info.	HBTRC	Suicide and Cause of death-Asphyxiation/suicide
S10	AN07617	Scz	77	M	25	BA 8	No Info.	Zyprexa 25 mg, Haloperidol-2 mg, Risperidone 4 mg and Olanzapine 20 mg once daily	No Info.	HBTRC	Natural and Cause of death-Aspiration Pneumonia
S11	AN08194	Scz	60	M	22	BA 8	No Info.	Zyprexa 30 mg and Invega 3 mg -once daily	No Info.	HBTRC	Natural and Cause of death-Atherosclerotic Heart disease
S12	AN05617	Scz	70	F	37	BA 8	No Info.	Mellaril 100 mg in the morning and 200 mg at night	No Info.	HBTRC	Natural
S13	AN09659	Scz	64	F	19	BA 8	No Info.	Zyprexa 20 mg-once daily	No Info.	HBTRC	Natural and Acute respiratory failure
S14	AN12657	Scz	56	M	24	BA 8	30	Risperdal 2 mg and Zyprexa 10 mg-twice daily	No Info.	HBTRC	Natural
S15	AN11287	Scz	49	M	8	BA 8	16	Clozaril 100 mg- 1 pill in the a.m and 2 in the p.m., Fluphenazine 10 mg-twice daily	No Info.	HBTRC	Natural
S16	AN10450	Scz	65	F	30	BA 8	14	Zyprexa 5 mg-once daily	No Info.	HBTRC	Natural and Chronic Obstructive Pulmonary Disease
S17	AN02738	Scz	85	F	13	BA 8		No Info.	No Info.	HBTRC	Natural
S18	AN13227	Scz	60	F	17	BA 8	20	Haloperidol 20 mg, Eskalith CR 450 mg-once daily	No Info.	HBTRC	Natural
S19	AN00216	Scz	63	M	26	BA 8	20s	Navane 10 mg-twice daily	No Info.	HBTRC	Natural
S20	AN18085	Scz	76	F	16	BA 8	27	Haloperidol 50 mg, Resperidone 0.25 mg-once daily	No Info.	HBTRC	Natural
C1	AN15948	Control	56	M	23	BA 8	N/A	None	No Info.	HBTRC	Natural and Cause of death-Cardiac Arrest
C2	AN10477	Control	46	F	29	BA 8	N/A	None	No Info.	HBTRC	Natural and Cause of death-Acute Dysrhythmia
C3	AN17188	Control	65	F	27	BA 8	N/A	None	No Info.	HBTRC	Natural
C4	AN01077	Control	58	F	19	BA 8	N/A	None	No Info.	HBTRC	Natural
C5	AN02883	Control	58	M	27	BA 8	N/A	Treated for Depression	No Info.	HBTRC	Accidental
C6	AN15717	Control	60	M	30	BA 8	N/A	None	No Info.	HBTRC	Natural
C7	AN04660	Control	66	M	23	BA 8	N/A	None	No Info.	HBTRC	Natural
C8	AN14913	Control	79	F	15	BA 8	N/A	None	No Info.	HBTRC	Natural
C9	AN04122	Control	84	F	18	BA 8	N/A	None	No Info.	HBTRC	Natural and Chronic Obstructive Pulmonary Disease
C10	AN05874	Control	63	M	24	BA 8	N/A	None	No Info.	HBTRC	Natural
C11	AN12916	Control	77	M	25	BA 8	N/A	None	No Info.	HBTRC	Natural and Cause of death-Cardiac Arrest
C12	AN15414	Control	59	M	23	BA 8	N/A	None	No Info.	HBTRC	Natural and Cause of death-Cardiopulmonary Arrest secondary to Abdominal Aortic Aneurysm
C13	AN12137	Control	31	M	33	BA 8	N/A	None	No Info.	HBTRC	Accident
C14	AN03874	Control	71	F	25	BA 8	N/A	None	No Info.	HBTRC	Natural and Chronic Obstructive Pulmonary Disease
C15	AN11937	Control	63	F	24	BA 8	N/A	None	No Info.	HBTRC	Natural
C16	AN01235	Control	52	M	8	BA 8	N/A	None	No Info.	HBTRC	Natural
C17	AN12203	Control	75	F	17	BA 8	N/A	None	No Info.	HBTRC	Natural
C18	AN03389	Control	69	F	19	BA 8	N/A	None	No Info.	HBTRC	Natural
C19	AN17166	Control	60	M	22	BA 8	N/A	None	No Info.	HBTRC	Natural
C20	AN04969	Control	60	F	22	BA 8	N/A	None	No Info.	HBTRC	Natural
C21	BBN_2347	Control	41	M	49	BA 11, 12	N/A	None	6,4	MRCE	Dilated cardiomyopathy
C22	BNN_2389	Control	37	F	46	BA 11, 12	N/A	None	6,2	MRCE	Multi-systemic failure, internal bleeding, liver failure
C23	BBN_2444	Control	37	M	63	BA 11, 12	N/A	None	6,4	MRCE	Ischaemic heart disease
C24	BBN_2501	Control	50	M	63	BA 11, 12	N/A	None	6,2	MRCE	Ischaemic heart disease
C25	BBN_2504	Control	70	M	96	BA 11, 12	N/A	None	6,3	MRCE	Coronary thrombosis, ischaemic heart disease
C26	BBN_2515	Control	69	M	35	BA 11, 12	N/A	None	6,0	MRCE	Ischaemic & hypertensive heart disease,
C27	BBN_2517	Control	36	M	41	BA 11, 12	N/A	None	6,4	MRCE	Cardiomegaly, ischaemic heart disease
C28	BBN_2525	Control	43	M	44	BA 11, 12	N/A	None	6,1	MRCE	Ischaemic heart disease
C29	BBN_2530	Control	51	M	52	BA 11, 12	N/A	None	6,3	MRCE	Ischaemic heart disease
C30	BBN_2537	Control	43	M	53	BA 11, 12	N/A	None	6,3	MRCE	Sudden cardiac death
C31	BBN_2568	Control	42	F	86	BA 11, 12	N/A	None	6,0	MRCE	Pulmonary congestion
C32	BBN-2580	Control	42	M	61	BA 11, 12	N/A	None	6,4	MRCE	Ischaemic heart disease
S21	BBN_2454	Scz	50	M	75	BA 11, 12	46	Flupentixol 30mg injection every 2 weeks	6,1	MRCE	Acute combined morphine, methadone, diazepam and alcohol toxicity
S22	BBN_2458	Scz	41	M	41	BA 11, 12	No Info.	None	6,4	MRCE	External asphyxia by inhalation of helium (suicide)
S23	BBN_2460	Scz	42	M	46	BA 11, 12	No Info.	Flupentixol 60mg injection monthly	6,4	MRCE	Suspension by ligature, increased alcohol intake
S24	BBN_2462	Scz	40	F	73	BA 11, 12	27	None	6,5	MRCE	Intra-cerebral haemorrhage (stroke), rupture of intra-cerebral artery, hypertension
S25	BBN_2496	Scz	69	M	67	BA 11, 12	19	None	6,1	MRCE	Hypertensive heart disease
S26	BBN_2562	Scz	36	M	50	BA 11, 12	35	Olanzapine, Amisulpride	6,3	MRCE	Cardiac arrhythmia, presumed drug toxicity
S27	SWED-001	SCZ	23	F	26	BA 8, 9	20	Risperidone 6 weeks of meds left	6,7	UMMC	Suicide
S28	SWED-002	Schizoaffective	27	M	20	BA 8, 9	20	Rx NORTRIPTYLINE 1 day before,fluphenazine,diphenyhydramine	6,6	UMMC	Suicide
S29	SWED-003	Scz	23	M	19	BA 8, 9	19	loxapine,benzotropine, enalapril (HTN),duramed	6,7	UMMC	Suicide
S30	SWED-004	Scz	35	F	15	BA 8, 9	35	thioridizine,dyazide (HTN),furosemide , synthroid .75 mg	6,7	UMMC	Suicide
S31	SWED-005	Scz	45	M	15	BA 8, 9	early 20s	THIOTHIXENE,DIPHENYHYDRAMINE	6,7	UMMC	Suicide
S32	SWED-006	Scz	51	M	18	BA 8, 9	36	LOXAPINE,TRIFLUORPERAZINE,CLONAZEPAM,BENZTROPINE	6,8	UMMC	Suicide
S33	SWED-007	Scz	59	F	14	BA 8, 9	48	Nothing listed	6,1	UMMC	Suicide
S34	SWED-008	Schizoaffective	41	F	26	BA 8, 9	30	RISPERIDONE,PERPHENAZINE,CLONAZEPAM, IMIPRAMINE,ALPRAZOLAM,TRIHEXYPHENIDYL	6,5	UMMC	Suicide
S35	SWED-009	Schizoaffective	41	F	74	BA 8, 9	~35 or 36	Zoloft, doxepin, xanax, cardizem, haldol, visteril, trazedone, celebrex, paxil	6,6	UMMC	Suicide
S36	SWED-010	Schizoaffective	42	M	15	BA 8, 9	31	TOD:Wellbutrin, Depakote, Seroquel, Lisinopril, Insulin Past Hx: Zyprexa, Haldol, Cogentin, Celexa, Lexapro, Geodon, Hydroxyzine	6,1	UMMC	Suicide
S37	SWED-011	Scz	32	M	10	BA 8, 9	27	Hx:haloperidol,benztropine	6,8	UMMC	Suicide
S38	SWED-012	Schizoaffective	34	M	6	BA 8, 9	33	thioridizine year prior	6,9	UMMC	Suicide
S39	SWED-013	Scz	31	F	29	BA 8, 9	15-17	MESORIDAZINE,FLUPHENAZINE,Li++,chlorprom,molindone, perphen,haloperidol,AMANTADINE,benztropine	6,1	UMMC	Myocardial infarct
S40	SWED-014	Scz	29	M	30	BA 8, 9	23	ATIVAN, HALDOL, REMERON, Zyprexa	6,8	UMMC	Acute pulmonary thromboembolism
S41	SWED-015	Scz	26	M	17	BA 8, 9	17	HALDOL; DIAMOX; HYDROCHLOROTHYIAZIDE; GABAPENTIN, METOPROLOL, LANSOLPRAZOLE, OLANZAPINE	6,3	UMMC	Hypertrophic cardiomyopathy
S42	SWED-016	Scz	43	F	21	BA 8, 9	27	TRIFLUOPERZINE,T4,prednisone,theophylline	6,5	UMMC	Malignant cerebral infarct
S43	SWED-017	Scz	43	M	16	BA 8, 9	Unknown	fluphenazine Hx:thiothixene	6,8	UMMC	Homicide
S44	SWED-018	Scz	47	M	16	BA 8, 9	~22-23	chlorpromazine	6,6	UMMC	Pulmonary Disease
S45	SWED-019	Scz	64	F	25	BA 8, 9	~42-43	FLUPHENAZINE,BENZTROPINE,BISACODYL,CISAPRIDE	6,7	UMMC	Heart disease
S46	SWED-020	Scz	45	F	6	BA 8, 9	20	LOXAPINE,AMOXAPINE,RISPERIDONE,BENZTROPINE,CARBAMAZEPINE	6,6	UMMC	Heart disease
S47	SWED-021	Scz	39	F	6	BA 8, 9	30	TRAZODONE,FLUOXETINE,BUPROPION,THIORIDAZINE,risperidone	6,8	UMMC	Chronic ehanol abuse with severe hepatosteatosis & hepatomegaly
S48	SWED-022	Scz	44	M	21	BA 8, 9	16	Per sister had been rxd: Cador 20 mg qd, Lanoxin, 250 mg, Dioxan 80 mg qd, Monophil 40 mg, Furosemid, Clonodine. Per CCCO records: Zyprexa, Lasix, Doxyclicline, ASA, Altace, Coreg, Heparin, Succinyl, Choline, Klor-Con, aerosal tx, possibly Diovan (per MD records) Unsure if taking	6,6	UMMC	Dilated cardiomyopathy due to hypertensive cardiovascular disease
S49	SWED-023	Scz	48	F	18	BA 8, 9	31	CHLORPROMAZINE 100mg	5,7	UMMC	Heart disease
S50	SWED-024	Scz	40	M	12	BA 8, 9	28	Fx noncompliant: coreg, lasix, spirolactone, solumedrol, diamox, lovenex, zinacef, zithromax, advair, aniphyl, digoxin, lisinopril, xanax, seroquel. Hx: loxitane, zyprexa, respirdal, haldol cogentin.	6,5	UMMC	Hypertensive atherosclerotic cardiovascular disease along with another condition of chronic obstructive pulmonary disease.
S51	SWED-025	Scz	34	M	19	BA 8, 9	22	meperidine,diazepam,DOXEPIN	6,8	UMMC	Accidental overdose
S52	SWED-026	Scz	65	F	22	BA 8, 9	late 20's - early 30's	Coumadin, Lisinopril Xanax (check med recs and CCCO)	6,6	UMMC	Dilated cardiomyopathy due to hypertensive arteriosclerotic
S53	SWED-027	Scz	58	M	24	BA 8, 9	~28	CHLORPROMAZINE, GLYBURIDE, QUETIAPINE, RISPERIDONE, HYDROXYZINE	5,9	UMMC	acute bronchopneumonia, severe coronary sclerotic heart disease
S54	BBN_16927	Scz	67	M	24	BA 8, 9	No Info.	Stelazine	No info	LNDBB	Myocardial infarction
S55	BBN_17035	Scz	87	F	7	BA 8, 9	No Info.	No info	No info	LNDBB	Bronchopneumonia
S56	BBN_00000	Scz	45	M	45	BA 8, 9	No Info.	No info	No info	LNDBB	Intra-abdominal sepsis
S57	BBN_16682	Scz	75	F	50	BA 8, 9	No Info.	Trifluoperazine	No info	LNDBB	Cerebrovascular accident (stroke)
S58	BBN_16685	Scz	35	M	67	BA 8, 9	No Info.	Flupenthixol	No info	LNDBB	Bronchopneumonia
S59	BBN_16744	Scz	49	M	24	BA 8, 9	No Info.	Chlorpromazine	No info	LNDBB	Myocardial infarction
S60	BBN_16735	Scz	34	F	23	BA 8, 9	No Info.	No info	No info	LNDBB	Acute pulmonary oedema
S61	BBN_16743	Scz	49	F	100	BA 8, 9	No Info.	No info	No info	LNDBB	Perforated duodenal ulcer
S62	BBN_16753	Scz	70	F	100	BA 8, 9	No Info.	Trifluoperazine	No info	LNDBB	Bronchopneumonia
S63	BBN_16817	Scz	87	M	48	BA 8, 9	No Info.	Stelazine	No info	LNDBB	Bronchopneumonia
S64	BBN_16783	Scz	32	F	46	BA 8, 9	No Info.	Chlorpromazine	No info	LNDBB	Pulmonary embolus
S65	BBN_16827	Scz	84	F	42	BA 8, 9	No Info.	No info	No info	LNDBB	Bronchopneumonia
S66	BBN_16580	Scz	62	M	48	BA 8, 9	No Info.	No info	No info	LNDBB	Ischaemic heart disease
S67	BBN_16617	Scz	51	M	44	BA 8, 9	No Info.	No info	No info	LNDBB	Myocardial infarction
S68	BBN_16641	Scz	46	M	38	BA 8, 9	No Info.	Sulpiride	No info	LNDBB	Multiple injuries
S69	BBN_16639	Scz	62	M	36	BA 8, 9	No Info.	No info	No info	LNDBB	Pulmonary tuberculosis
S70	BBN_16646	Scz	71	F	65	BA 8, 9	No Info.	Chlorpromazine	No info	LNDBB	Pulmonary embolus
S71	BBN_16624	Scz	31	M	27	BA 8, 9	No Info.	Chlorpromazine	No info	LNDBB	Multiple organ failure
S72	BBN_16889	Scz	79	M	48	BA 8, 9	No Info.	Promazine	No info	LNDBB	Cardiac arrest
S73	BBN_16866	Scz	76	F	97	BA 8, 9	No Info.	Haloperidol	No info	LNDBB	Bronchopneumonia
C33	BBN_18401	Control	82	M	47	BA 8, 9	N/A	No info.	No info	LNDBB	Congestive cardiac failure, aortic stenosis
C34	BBN_16236	Control	89	F	41	BA 8, 9	N/A	No info.	No info	LNDBB	Aortic sclerosis
C35	BBN_4568	Control	90	F	50	BA 8, 9	N/A	No info.	No info	LNDBB	No info
C36	BBN_16291	Control	81	M	18	BA 8, 9	N/A	No info.	No info	LNDBB	No info.
C37	BBN_15719	Control	59	M	50	BA 8, 9	N/A	No info.	No info	LNDBB	Non-smallcell lung cancer
C38	BBN_15765	Control	55	M	24	BA 8, 9	N/A	No info.	No info	LNDBB	Syringomyelia
C39	BBN_15790	Control	40	M	40	BA 8, 9	N/A	No info.	No info	LNDBB	CA liver, renal and respiratory failure
C40	BBN_15805	Control	52	F	44	BA 8, 9	N/A	No info.	No info	LNDBB	Carcinoma of the lung
C41	BBN_15609	Control	68	F	9	BA 8, 9	N/A	No info.	No info	LNDBB	Systemic lupus erythematosus
C42	BBN_15611	Control	60	M	35	BA 8, 9	N/A	No info.	No info	LNDBB	Cardiac arrest
C43	BBN_16429	Control	68	M	53	BA 8, 9	N/A	No info.	No info	LNDBB	Ischaemic heart disease
C44	BBN_15621	Control	61	M	53	BA 8, 9	N/A	No info.	No info	LNDBB	Cardiac arrest
C45	BBN_16253	Control	43	F	43	BA 8, 9	N/A	No info.	No info	LNDBB	Severe obesity
C46	BBN_16507	Control	33	F	96	BA 8, 9	N/A	No info.	No info	LNDBB	Pulmonary embolus
C47	BBN_16432	Control	67	F	24	BA 8, 9	N/A	No info.	No info	LNDBB	Myocardial infarction
C48	BBN_16485	Control	76	F	63	BA 8, 9	N/A	No info.	No info	LNDBB	Bronchopneumonia
C49	BBN_16525	Control	77	F	29	BA 8, 9	N/A	No info.	No info	LNDBB	Pulmonary embolus
C50	BBN_16785	Control	37	M	27	BA 8, 9	N/A	No info.	No info	LNDBB	Acute necrotic pancreatitis
C51	BBN_16678	Control	49	M	44	BA 8, 9	N/A	No info.	No info	LNDBB	Pulmonary oedema
C52	BBN_16523	Control	49	M	32	BA 8, 9	N/A	No info.	No info	LNDBB	Coronary artery occlusion

HBTRC - Harvard Brain Tissue Resource Center
MRCE - MRC UK Brain Bank Networks
UMMC - University of Mississippi Medical Center Brain Collection
LNDBB - London Neurodegenerative Disease Brain Bank
PMI - Post Mortem Interval
