# Supplementary material for: Exploring autoantibody signatures in brain tissue from patients with severe mental illness
Source: Transl Psychiatry. 2020 Nov 18;10:401. doi: 10.1038/s41398-020-01079-8 (PMC7676257; doi:10.1038/s41398-020-01079-8)
Supplement: Supplementary file 7 — Supplementary Table 3 [file 41398_2020_1079_MOESM7_ESM.pdf]

Gene Name	Protein name	Uniprot ID	antigen #	PrEST seq (aa)
ADAMTS4	A disintegrin and metalloproteinase with thrombospondin motifs 4	O75173	HPRR3760736	VEGLTVQYLGQAPELLGGAEPGTYLTGTINGDPESVASLHWDGGALLGVLQYRGAELHLQPLEGGTPNSAGGPGAHILRRKSPASGQGPMCN
ADGRF3	Adhesion G-protein coupled receptor F3	Q8IZF5	HPRR1600059	SPSDLLTLLSTMKYVAKVVAEARIQLDRRALKNLLIATDKVLDMDTRSLWTLAQAQKPWAGSTLLLAVETLACSLCPQDHPFAFSLPNVLLQSQLFGPTFPADYSISFPTRPPLQAQIPRHSLAPLVRNGTEISITS
ATXN10	Ataxin-10	Q9UBB4	HPRR3730524	KHPESEWPFLIITDLFLKSPELVQAMFPKLNNQERVTLLDLMIAKITSDEPLTKDDIPVFLRHAELIASTF
C9orf47	Uncharacterized protein C9orf47	Q6ZRZ4	HPRR3420719	LWAPGFCTWSSPDEDKVWRP
CCT8L2	T-complex protein 1 subunit theta-like 2	Q96SF2	HPRR3200008	EGIINVAQEGVWDTLIVKAQGFRAVAEVVLQLVTVDEIVVAKK
CLHC1	Clathrin heavy chain linker domain-containing protein 1	Q8NHS4	HPRR3400238	LTKYMKHLEDKYAEIKQAMLIKYVPAQRKADLDEEMIVLLKRRDVAENLNKKLQFCHQRLQIISQALSSWVKSDMSSPFQDFVEQIQKTKY
DOK5	Docking protein 5	Q9P104	HPRR3720425	YWQHITRQHSTGQLYRLQDVSSPLKLHRTETFPAYRSEH
LSM10	U7 snRNA-associated Sm-like protein LSm10	Q969L4	HPRR2551844	MAVSHSVKERTISENSLIILLQGLQGRVTTVDLRDESVAHGRIDNVDAFMNIRLAKVTYT
LTB	Lymphotoxin-beta	Q06643	HPRR3450260	GGLVTETADPGAQAQQGLGFQKLPEEEPETDLSPGLPAAHLIGAPLKGQGLGWETTKEQAFLTSGTQFSD
RYK	Tyrosine-protein kinase RYK	P34925	HPRR1840145	SMKRIELDDSISASSSSQGLSQPSTQTTQYLRADTPNNATPITSSLGYPTLRIEKNDLRSVTLLEAKGKVKDIAISRERITLKDVLQEGTFGRIFHGILIDEKDPNKEKQAFVKTVKDQASEIQVTMMLTESCKLRGLHHRNLLPITH
SPATA31A1	Spermatogenesis-associated protein 31A6	Q5VVP1	HPRR3340120	KVKFQLERDPCPHLGQILGETPQNLSRDMKSFPRKVLGVTSEELERNLRKPLRSDSGSDLLRCTERTHIENILKAHMGRNLGQTNEGLIPVRVRRSWLAVNQALPVSN
STAT5B	Signal transducer and activator of transcription 5B	P51692	HPRR3290111	KTQTKFAATVRLLVGGKLNVHMNPPQVKATIISEQQAKSLLKNENTRN
TMCO1	Calcium load-activated calcium channel	Q9UM00	HPRR2850351	RQNIQKILGLAPSRAATKQAGGFLGPPPPSGKFS
URB2	Unhealthy ribosome biogenesis protein 2 homolog	Q14146	HPRR1500008	PEGAVVAQLFEVIHLALGHYLLILQQQVNPRRAFGDVTAHLLQPCLVLRHLLSGGTWTQAGQGQLRQVLSRDIRSQIEAMFRGGIFQPELLSSYKEGLLDQQQGDVKTGAMKNLLAPMDTVLNRLVDAGYCAASL
